# Draft Genome Sequence of Dihydroxyacetone-Producing *Gluconobacter thailandicus* Strain NBRC 3255

**DOI:** 10.1128/genomeA.00118-13

**Published:** 2013-04-11

**Authors:** Minenosuke Matsutani, Erika Kawajiri, Toshiharu Yakushi, Osao Adachi, Kazunobu Matsushita

**Affiliations:** Department of Biological Chemistry, Faculty of Agriculture, Yamaguchi University, Yamaguchi, Japan

## Abstract

Here, we report the draft genome sequence of the acetic acid bacterium *Glucnobacter thailandicus* strain NBRC 3255. The draft genome sequence is composed of 109 contigs in 3,305,227 bp and contains 3,225 protein-coding genes. Two paralogous sets of *sldAB* operons, which are responsible for dihydroxyacetone production from glycerol, were identified.

## GENOME ANNOUNCEMENT

Acetic acid bacteria (AAB) are obligate aerobic, Gram-negative, rod-shaped bacteria that are well known for their potential to incompletely oxidize a wide variety of sugars, alcohols, and polyols. Members of the genus *Gluconobacter*, which belongs to the group of acetic acid bacteria, can oxidize a wide range of alcohols, sugars, and sugar alcohols and can accumulate a large amount of the corresponding oxidized products in culture medium (1). *Gluconobacter* strains are also known as interesting organisms for industrial applications. Industrial fermentation processes, such as the production of l-sorbose (vitamin C synthesis), 6-amino-l-sorbose, and dihydroxyacetone, are carried out by members of this genus.

Here, we report the draft genome sequence of *Gluconobacter thailandicus* strain NBRC 3255, which was isolated from a strawberry in Japan. The genome of *G. thailandicus* NBRC 3255 was sequenced with the next-generation sequencing platform Illumina HiSeq 2000 and generated 16,059,176 paired-end reads. The draft genome sequence was assembled using the Velvet assembler v1.1.02 (2), and 972× genome coverage resulted in a final assembly of 3,305,227 bp with a G+C content of 56.2% and an *N*_50_ length of 115,646 bp. Protein-coding gene prediction was performed by Glimmer 3.02 with self-training Dataset (3), and tRNAs and rRNAs were predicted using ARAGORN and RNAmmer, respectively (4, 5). Functional annotation of the predicted genes was performed by BLASTp searching of the nonredundant (NR) database (6). The draft genome sequence of *G. thailandicus* NBRC 3255 contains 109 contigs, including 76 large contigs (>1,000 bp). A total of 3,225 protein-coding genes were identified. Fifty-three tRNA genes and 3 rRNA genes were identified.

To investigate the genomic characteristics of this strain, comparative analysis with the genome of *Gluconobacter oxydans* 621H was performed. Comparative genome analysis identified several coding genes that are unique to the genome of *G. thailandicus* NBRC 3255 (7). A unique orphan gene of the *adh* subunit I was identified, which is also conserved in the genome of *Acetobacter pasteurianus* IFO3283-01 (8). Besides genes for NADH dehydrogenase II (two paralogs), a proton-pumping NADH:ubiquinone oxidoreductase (NADH dehydrogenase I) operon (NBRC3255_2101 to NBRC3255_2113) was identified. The gene repertories for proteins containing a PQQ domain were also compared. NBRC 3255 lacks the PQQ-dependent dehydrogenase 1, while two paralogous sets of *sldAB* operons (NBRC3255_0025 to NBRC3255_0026 and NBRC3255_0235 to NBRC3255_0236) were identified.

One of the two paralogs, the NBRC3255_0235 operon, is responsible for dihydroxyacetone production from glycerol as a glycerol dehydrogenase (9), and NBRC 3255 was shown to assimilate dihydroxyacetone (10). Although Prust et al. suggested that the *G. oxydans* 621H strain assimilates dihydroxyacetone (7), we found that the strain fails to metabolize it (E. Kawajiri, M. Matsutani, T. Yakushi, O. Adachi, and K. Matsushita, unpublished data). Comparative genomics identified that NBRC 3255 has a second dihydroxyacetone kinase gene (NBRC3255_2003), which would be crucial to dihydroxyacetone assimilation in NBRC 3255.

### Nucleotide sequence accession numbers. 

The draft genome sequence for *G. thailandicus* NBRC 3255 has been deposited in DDBJ/EMBL/GenBank under the accession no. BAON00000000. The version described in this paper is the first version, accession no. BAON01000000.
